# Metal Oxide Oxidation Catalysts as Scaffolds for Perovskite Solar Cells

**DOI:** 10.3390/ma13040949

**Published:** 2020-02-20

**Authors:** Peter J. Holliman, Arthur Connell, Eurig W. Jones, Christopher P. Kershaw

**Affiliations:** College of Engineering, Bay Campus, Swansea University, Swansea SA1 8EN, UK; arthur.connell@swansea.ac.uk (A.C.); eurig.w.jones@swansea.ac.uk (E.W.J.); c.p.kershaw@swansea.ac.uk (C.P.K.)

**Keywords:** organolead Perovskite, low temperature sintering, mesoporous scaffold, oxidation catalyst

## Abstract

Whilst the highest power conversion efficiency (PCE) perovskite solar cell (PSC) devices that have reported to date have been fabricated by high temperature sintering (>500 °C) of mesoporous metal oxide scaffolds, lower temperature processing is desirable for increasing the range of substrates available and also decrease the energy requirements during device manufacture. In this work, titanium dioxide (TiO_2_) mesoporous scaffolds have been compared with metal oxide oxidation catalysts: cerium dioxide (CeO_2_) and manganese dioxide (MnO_2_). For MnO_2_, to the best of our knowledge, this is the first time a low energy band gap metal oxide has been used as a scaffold in the PSC devices. Thermal gravimetric analysis (TGA) shows that organic binder removal is completed at temperatures of 350 °C and 275 °C for CeO_2_ and MnO_2_, respectively. By comparison, the binder removal from TiO_2_ pastes requires temperatures >500 °C. CH_3_NH_3_PbBr_3_ PSC devices that were fabricated while using MnO_2_ pastes sintered at 550 °C show slightly improved PCE (η = 3.9%) versus mesoporous TiO_2_ devices (η = 3.8%) as a result of increased open circuit voltage (V_oc_). However, the resultant PSC devices showed no efficiency despite apparently complete binder removal during lower temperature (325 °C) sintering using CeO_2_ or MnO_2_ pastes.

## 1. Introduction

Scaling solar cell manufacturing requires a switch from batch to roll-to-roll (R2R) processing to accelerate throughput [1]. Third generation photovoltaics, such as perovskite solar cells (PSC), offer the potential of R2R manufacturing because these are solution processable and are, therefore, printable devices [2]. However, R2R processing requires flexible substrates; e.g., metal foil or transparent conducting oxide (TCO) coated plastic film. Thus, whilst the most widely used form of TCO-coated glass (fluoride-doped tin oxide—FTO) can withstand 500 °C, these substrates are rigid. By comparison, whilst metal foils are flexible, they rapidly oxidize at this temperature, whilst plastic films (e.g., PET) are limited to ca. 120 °C when under tension, which is necessary for R2R operation. As such, there is a need to lower the manufacturing temperature of perovskite solar cell devices.

A key reason for the global interest in PSC devices is the rapid increase in device efficiencies, leading to a certified world record power conversion efficiency (PCE) of 25.2% [3]. The highest efficiency devices are fabricated using a mesoporous metal oxide scaffold sintered at high temperature >500 °C [4,5]. However, for lower temperature processing (<200 °C), the highest PCE reported for mesoporous metal oxide PSCs is 18.2% [6]. By comparison, low temperature fabrication of non-scaffold PSCs i.e., planar devices, has resulted in PCE >20% [7,8]. Not only does lower temperature processing broaden the range of substrates available to include lower cost substrates, but it also lowers PSC embodied energy [6,9].

For mesoporous PSCs the metal oxide is deposited from a paste containing solvent and organic binders to control the rheology and film morphology before and after deposition [4,5]. High temperature processing is required to burn off the organic binders and sinter the metal oxide particles together [10,11]. Schulze et al. and Brown et al. removed the organic binders while using UV treatment [6,9]. However, our previous work focused on dye sensitized solar cell (DSC) devices, we reported lower temperature binder removal during device fabrication by mixing a metal peroxide oxidation catalyst into a TiO_2_ working electrode precursor paste [11]. Here, we have further explored the use of oxidation catalysts by fabricating PSCs while using CeO_2_ and MnO_2_ mesoporous scaffolds. CeO_2_ is well established as an oxidation catalyst, as is discussed in several review articles [12,13]. It has also been reported as an ETL for planar PSCs fabricated at low temperature [14,15]. Xie and Deng et al. reported a PCE of 14.3% for n-i-p PSCs while using a CeO_2_ ETL that was deposited from a sol-gel precursor and sintered at 150°C. The PCE was increased to 17.0% by adding an organic ETL as an interlayer between the CeO_2_ and perovskite to reduce trap assisted recombination at the interface [14]. Chen et al. reported a champion PCE of 17.0% for p-i-n PSC devices that were fabricated while using CeO_2_ as an ETL sintered at 100°C. In their work, the authors state that the CeO_2_ ETL is more compact and it offers better moisture resistance in comparison to organic ETLs. PSC devices fabricated while using CeO_2_ have also been reported to exhibit improved stability in comparison to devices that were fabricated with an organic ETL [15]. MnO_2_ is also a well-established oxidation catalyst [16,17]. To the best of our knowledge, the use of narrow band gap metal oxides, including MnO_2_, as ETLs in PSC devices has not been reported before. For n-i-p devices, the reason being is thought to be that a low band gap ETL might absorb photons that would decrease the photocurrent generated by the perovskite absorber. However, MnO_2_ has been used to improve the performance of photovoltaic devices. Lee et al. reported enhanced electron injection and decreased recombination in organic light emitting diodes while using MnO_2_ as an interlayer between the organic emitter and cathode [18]. Improved electron transport was also reported for DSC devices that were fabricated while using TiO_2_-MnO_2_ composite working electrodes [19]. MnO_2_ has also been reported as a low-cost counter electrode in DSC devices [20]. 

In this paper, we report the fabrication of methylamine lead tribromide PSC devices using either MnO_2_ or CeO_2_ mesoporous scaffold layers, which have been compared with mesoporous TiO_2_ scaffold devices. The thermal chemistry of the sintering process has been correlated with device data to show the importance of effective sintering to ensure complete binder removal and achieve stoichiometric metal oxide formation to achieve good device efficiencies. The world record device efficiency that is reported for this perovskite absorber is 10.4% [21].

## 2. Materials and Methods 

The materials were purchased from the following suppliers. TiO_2_ electron transport layer, Solaronix BL/SC, and FTO (10 Ω per square) substrates were purchased from Solaronix (Aubonne, Switzerland). TiO_2_ mesoporous scaffold paste, DSL18NR-T was purchased from Dyesol (Elanora, Australia). MnO_2_, CeO_2_, terpineol, and ethyl cellulose (48.0–49.5% (w/w) ethoxyl basis) were purchased from Sigma Aldrich (Dorset, UK). Methyl ammonium bromide (CH_3_NH_3_Br) was purchased from Sigma Aldrich (Dorset, UK) and lead bromide (PbBr_2_ 99.99%) was purchased from TCI Chemicals (Oxford, UK). The poly-3-hexylthiophene (P3HT) hole transport material (HTM) Mw = 54,200 was purchased from Ossila (Sheffield, UK). Bis(trifluoromethane) sulfonimide lithium salt (LiTFSI) and tertiary butyl pyridine (*t*BP) were purchased from Sigma Aldrich. The anhydrous solvents, including dimethylformamide (DMF), dimethylsulfoxide (DMSO), acetonitrile (CH_3_CN), chlorobenzene (ClBz), ethyl acetate (EtOAc), and ethanol (EtOH), were purchased from Sigma Aldrich (Dorset, UK). The solvents for cleaning and rinsing electrodes, including acetone and methanol (MeOH), were purchased from Fisher-Scientific (Loughborough, UK). 

CeO_2_ and MnO_2_ mesoporous scaffold pastes were prepared by adding 0.1 g of ethyl cellulose to 0.8 g of terpineol and stirring until the mixture appeared to be transparent (typical stirring times were 4 h at 800 rpm). To this mixture, 0.1 g of either CeO_2_ or MnO_2_ was added in *ca.* 0.025 g aliquots with 30 min stirring between additions to minimize metal oxide aggregation. 

For control devices, a solution of Solaronix BL/SC (100 µL) was spin coated onto pre-etched, acetone rinsed, and O_2_ plasma cleaned FTO substrates (2 cm × 2 cm) at 5000 rpm spin rate for 30 s while using an acceleration rate of 2000 rpm. This TiO_2_ layer was heated at 50 °C/min^−1^ to 550 °C and held for 1h. A 150 µL solution of DSL18NR-T mixed with EtOH (1:3 v/v) was spin coated onto this at 2000 rpm spin rate for 30 s using an acceleration rate of 500 rpm. This was heated at 10 °C/min^−1^ to 150 °C, 275 °C, 325 °C, and 450 °C (15 min dwell at each temperature), and finally sintered at 550 °C for 30 min to produce a mesoporous TiO_2_ film on an FTO substrate. For CeO_2_ and MnO_2_ PSC devices, the CeO_2_ and MnO_2_ pastes were mixed with EtOH (1:3 v/v) and processed as described for TiO_2_. However, for low temperature devices, the mesoporous films were heated at 10 °C/min^−1^ to 150 °C, 275 °C, 325 °C (15 min dwell at each temperature) and finally sintered at 325 °C for 30 min. 

Methylamine lead tribromide (CH_3_NH_3_PbBr_3_) perovskite absorber layers were prepared by mixing PbBr_2_ (1 g, 2.7 mM) with methylamine bromide (CH_3_NH_3_Br, 0.31 g, 2.7 mM) in anhydrous DMF (1.6 mL) and anhydrous DMSO (0.4 mL) for 4 h under N_2_ to create a perovskite precursor solution. This precursor solution was filtered through a 0.45 µm polytetrafluoroethylene (PTFE) filter (Sigma Aldrich, Dorset, UK) in ambient conditions. 100 µL of CH_3_NH_3_PbBr_3_ precursor solution was syringed onto a 2 cm × 2 cm the mesoporous film that had been pre-sintered on a FTO glass substrate. Perovskite films were formed by spin coating by ramping the spin coater to 1000 rpm (ramp rate 500 rpm) for 10 s, followed by a further 30 s at 4000 rpm (ramp rate 2000 rpm). During the final 5 s, 200 µL of anhydrous ethyl acetate was injected onto the sample, resulting in precipitation of the yellow CH_3_NH_3_PbBr_3_ perovskite. The films were annealed at 100 °C for 10 min in ambient conditions. The hole transport material (HTM) solution was P3HT (10 mg mL^−1^) in anhydrous chlorobenzene containing LiTFSI, where 10 µL of a stock solution was prepared while using 0.520 g of LiTFSI in 1 mL anhydrous CH_3_CN and *t*BP (10 µL). The HTM solution was gently heated to fully dissolve the components before filtering through a 0.45µm PTFE filter. 100 µL of HTM solution was deposited onto the annealed perovskite and then spin coated at 4000 rpm for 30s (ramp rate 2000rpm). The gold electrodes (ca. 80 nm) were thermally evaporated under vacuum of ~5 × 10^−6^ Torr at a rate of ~ 1 Å s^−1^.

Current density–voltage (*J-V*) curves for all devices were measured while using a 2400 Series Source Meter (Keithley Instruments, Farnell, Leeds, UK) under simulated AM 1.5 sunlight at 100mW cm^−2^ irradiance using an Abet Class AAB simulator (Sun 2000, Connecticut, US) that was calibrated with a ReRa solutions Si reference cell and with a PSC device active area of 0.15 cm^2^. X-ray diffraction data were measured using a Philips Bruker D8 (Cambridge, UK) discover instrument with a Cu source (λ = 1.54060 Å). Thermal gravimetric analysis and differential scanning calorimetry data were measured while using a LABSYS evo STA (Stockport, UK). The pastes were pre-dried at 100–110 °C for 2 h to evaporate the solvent to focus analysis on binder combustion. The samples were ramped at 10 °C min^−1^ under flowing air. The scanning electron microscopy images were measured using a Zeiss Evo MA25 (Cambridge, UK) high resolution scanning electron microscope with a LaB_6_ electron source (Cambridge, UK). 

To test perovskite stability, the CH_3_NH_3_PbBr_3_ films were deposited onto TiO_2_, CeO_2_, or MnO_2_ films, as described previously, these films were then subjected them to UV light (λ = 365 nm) for 24 h using a 6 Watt lamp (Analytikjena UVLS-26 EL Series, Jena, Germany), whilst also in a high humidity environment (>70%). Humidity was generated while using a GenRH Humidity generator (Surface Measurement Systems Ltd., Middlesex, UK) using de-ionized water. X-ray diffraction data of these films were analyzed before and after exposure to UV/humidity.

## 3. Results and Discussion

### 3.1. Material Characterization

**Figure 1:**
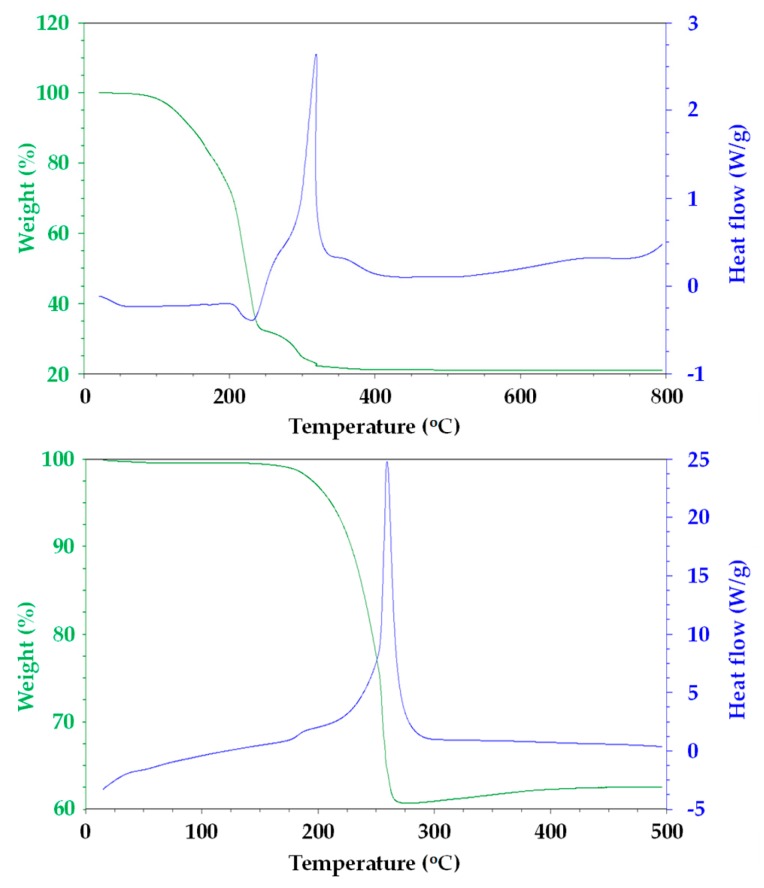
Thermal gravimetric and heat flow data from (top) CeO_2_ and (bottom) MnO_2_ pastes. Exotherm is up on the heat flow data.

Figure 1 shows TGA/DSC data for the residue of CeO_2_ and MnO_2_ pastes after solvent evaporation to focus on the key process of organic binder removal [10,11]. For CeO_2_, the majority of mass loss is complete by ca. 350 °C, after which the mass residue remains constant. The major mass loss from 250–350 °C is also associated with a strong exotherm (ca. 310 °C), being in line with a combustion process releasing CO_2(g)_ and heat, respectively. By comparison, the data for the MnO_2_ paste show a single mass loss from 175–275 °C. Again, this is associated with a strong exotherm in line with combustion taking place, but this feature is shifted to lower temperature (ca. 260 °C). However, very interestingly, after the combustion event, there is a small weight gain (ca. 3%) as the temperature is increased above 400 °C. We ascribe this to changes in the oxidation state of manganese ions within this metal oxide. Thus, during combustion, O_2_ from the surrounding air atmosphere will be consumed to convert the ethyl cellulose binder into CO_2(g)_. At the same time, at these elevated temperatures, it is likely that oxide ions from the MnO_2_ particles will be converted into O_2_ molecules by the simultaneous reduction of Mn^4+^ ions to Mn^2+^. However, the mass loss that is associated with this process will be coincident with the ethyl cellulose combustion and so is not resolvable in these TGA data. However, after ethyl cellulose, combustion is complete (ca. 275 °C), the Mn^2+^ ions will be re-oxidized by the additional O_2_ molecules present in the flowing air atmosphere. However, because there are no other thermal processes taking place at this point, the uptake of oxygen is observable as a mass increase in the TGA data. This has important consequences for perovskite devices made using MnO_2_ films that were sintered at different temperatures, because the presence of Mn^2+^ ions within the lattice will introduce new energy levels that can interfere with efficient device operation.

**Figure 2:**
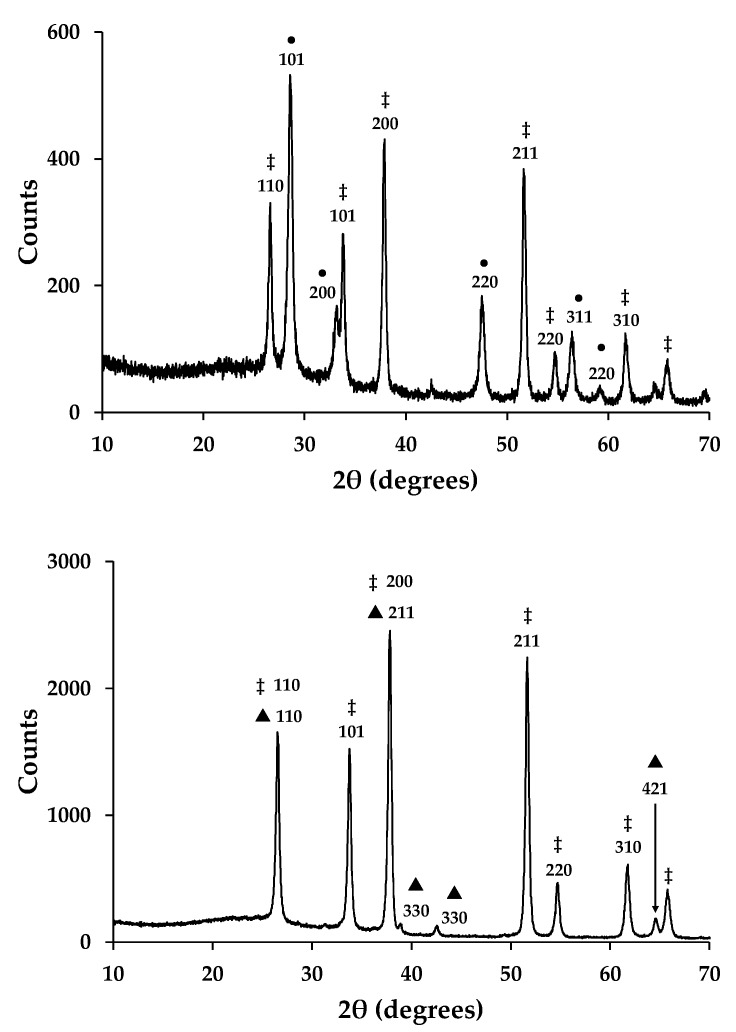
X-ray powder diffraction data for mesoporous films of (top) CeO_2_ and (bottom) MnO_2_ showing indexed diffraction peaks. Key: ‡ = SnO_2_, ● = CeO_2_, and ▲ = MnO_2_.

Figure 2 shows X-ray powder diffraction data for sintered, mesoporous metal oxide films. The data have been correlated with standard XRD patterns and they confirm the presence of cassiterite (SnO_2_-FTO JCPDS 00-001-0625 and 00-001-0657) in all samples. This is ascribed to the transparent conducting oxide (fluoride-doped tin oxide) that is present on the TEC glass used as the substrate for these metal oxide films. The data for the ceria film also confirm the presence of crystalline CeO_2_ particles (CeO_2_-JCPDS 00-001-0800 and JCPDS 00-034-0394). The data are also similar to the raw CeO_2_ powder, which suggests that the particles are randomly arranged within the mesoporous film and they do not show preferred orientation. By comparison, the data for the manganese oxide film confirm the presence of crystalline α-MnO_2_ particles [22]. Here, again there is no evidence for preferred orientation, which suggests that the MnO_2_ particles are randomly arranged in the mesoporous film.

**Figure 3:**
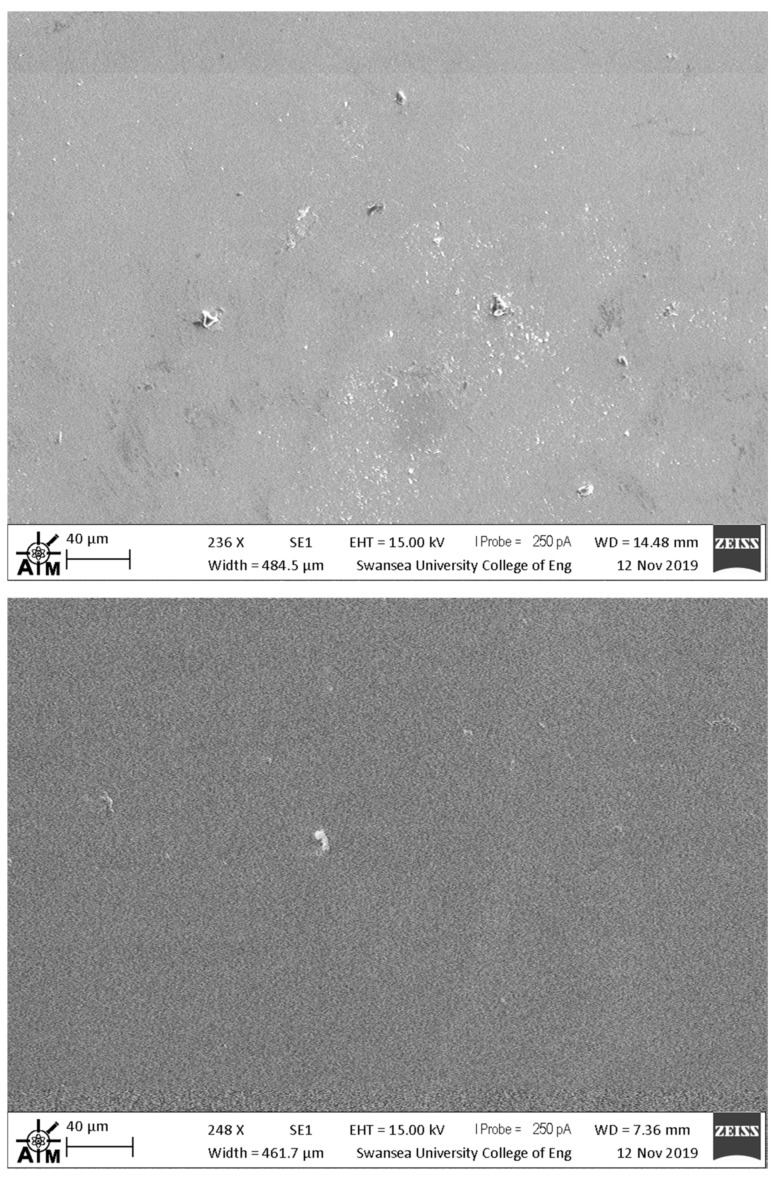
SEM images of mesoporous films of (top) CeO_2_ and (bottom) MnO_2_ films that were sintered at 550 °C on fluoride-doped tin oxide (FTO) glass.

Figure 3 shows plan view, scanning electron micrographs of CeO_2_ and MnO_2_ mesoporous films that were sintered at 550 °C. For the majority of the both films, the data show coherent surface coverage. However, both of the films also show larger particulates within the films which suggests that there were larger agglomerates present within the metal oxide pastes deposited onto the substrates. These larger particles would be expected to exert localized changes in device performance, although only over very small surface areas (< 10 μm). The SEM data also suggest that larger particles are present in the MnO_2_ film as compared to the CeO_2_ film. In parallel with this, the SEM data also suggest that the MnO_2_ film is more porous than the CeO_2_. In this context, when making repeat batches of metal oxide pastes, we did experience problems of reproducibility from batch to batch of devices, which results from the need to disperse pre-made MnO_2_ and CeO_2_ powders into solvent with the ethyl cellulose binder. To overcome this required significant mixing and homogenization to ensure consistency. For the majority of the surface, SEM analysis shows that the 550 °C-sintered CeO_2_ electrodes show more coherent surface coverage than those sintered at 325 °C. For MnO_2_ electrodes, the SEM analysis shows almost identical surface coverage for films that were sintered at 325 °C and 550 °C.

### 3.2. Perovskite Device Performance 

**Figure 4:**
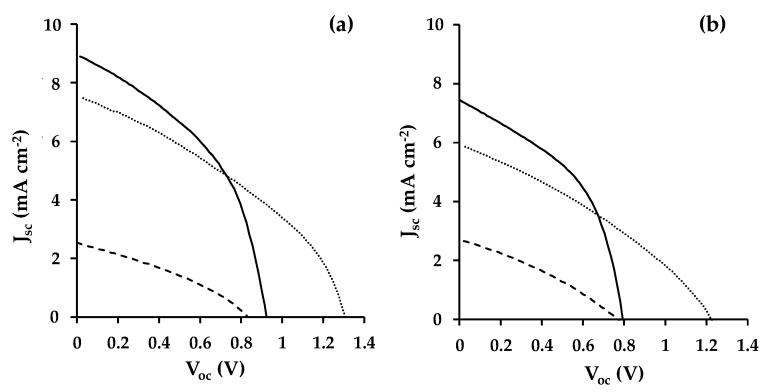
PSC I-V data scanned from (**a**) open circuit to short circuit and (**b**) short circuit to open circuit fabricated using metal oxide scaffolds consisting of TiO_2_ (solid line), CeO_2_ (dashed line), or MnO_2_ (dotted line).

Table 1 and Figure 4 show a summary of the I-V data that were measured for champion devices fabricated during this study. The lowest overall device efficiency (η ≈ 0.8%) was measured for devices that were fabricated while using CeO_2_ as a mesoporous scaffold mainly as a result of poor photocurrent generation (J_sc_ ≈ 2.7 mA cm^−2^). By comparison, the MnO_2_ PSC device showed a very similar PCE to the TiO_2_ control devices (ca. 3.8%). However, the data show a very substantial (ca. 400 mV) increase in open circuit voltage (V_oc_ = 1.34 V for MnO_2_ versus 0.95 V for TiO_2_). This was counteracted by lower photocurrent and FF for the MnO_2_ devices resulting in a similar overall device efficiency. In addition, the PCE for all devices was higher when measured from open circuit to short circuit. This type of hysteresis is routinely observed for perovskite solar cell devices and is mainly due here to a decrease of V_oc_ and J_sc_ when scanning from short circuit to open circuit (Figure 4). Using these I-V curve data, the hysteresis for TiO_2_, CeO_2_ and MnO_2_ devices has been calculated to be 25.6%, 8.8% and 32.4%, respectively. However, when considering the lower hysteresis that was observed for the CeO_2_ devices, it should be noted that the overall PCE for these devices is ca. 80% lower than for the TiO_2_ or MnO_2_ devices.

**Figure 5:**
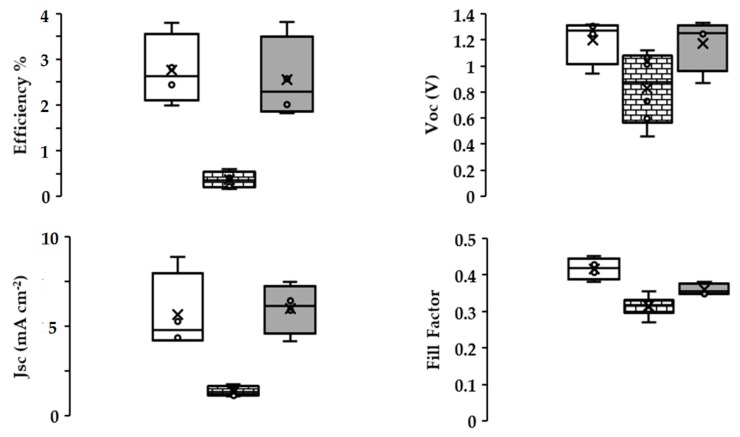
Box plots of (top left) device efficiency, (top right) V_oc_, (bottom left) normalized J_sc_ and (bottom right) fill factor. TiO_2_, CeO_2_, and MnO_2_ scaffold devices are represented by white, brick and grey fill, respectively. The data for each treatment are from 4 devices each with three pixels representing 12 measurements per sample.

**Figure 6:**
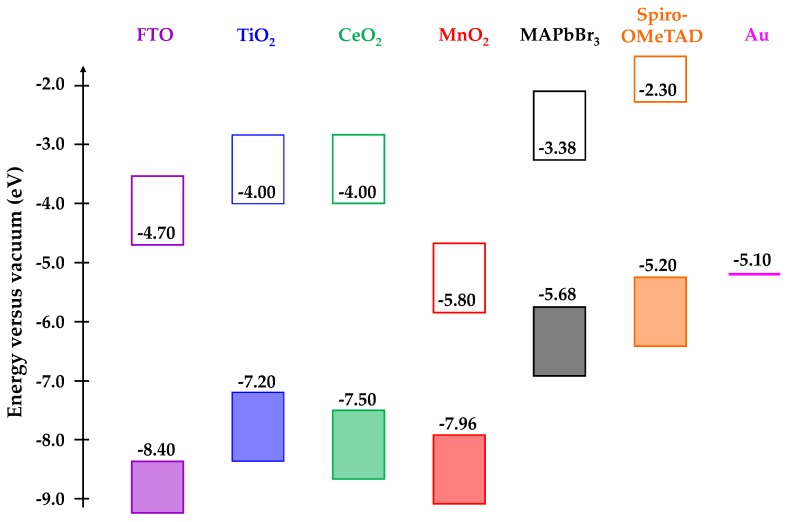
Energy level diagram for TiO_2_, CeO_2_, and MnO_2_ metal oxide scaffolds and CH_3_NH_3_PbBr_3_ (MAPbBr_3_) perovskite absorber showing conduction bands (clear boxes) and valence bands (shaded boxes). The spiro-OMeTAD hole transport material (HTM) and electrode materials-fluoride-doped tin oxide (FTO) and gold are shown for clarity.

Figure 5 shows the variation of device efficiency, V_oc_, normalized J_sc_, and fill factor obtained from open circuit to short circuit I-V scans for champion pixels measured from four replicate devices for each metal oxide scaffold type. The data show that the device efficiency and V_oc_ distribution are slightly greater for devices fabricated with MnO_2_ scaffolds when compared with TiO_2_-based devices. However, the J_sc_ and FF distribution showed the opposite trend with slightly great variation for TiO_2_ devices over MnO_2_. One explanation for these differences could be that the CH_3_NH_3_PbBr_3_ absorber might behave differently between the TiO_2_ and MnO_2_ devices. Thus, for TiO_2_, because the perovskite injects excited state electrons into the TiO_2_ scaffold, device efficiency is likely to be most affected by the perovskite-TiO_2_ interface and J_sc_ varies the most, because perovskite deposition and surface coverage is likely to be the biggest variable during device manufacture. However, for the MnO_2_ devices, there is such an offset in metal oxide/perovskite energy levels (Figure 6), it is unlikely that electron injection into the MnO_2_ occurs. Instead, the charge is most likely carried within the perovskite absorber itself, as was first reported by Snaith et al. for electrically insulating Al_2_O_3_-based perovskite devices [23]. By comparison, with the exception of V_oc_, the CeO_2_ devices gave less variable data, but that reflects the consistently poor CeO_2_ device performance. 

It is important to note that all of the devices were measured through the metal oxide film. Whilst the high band gaps of TiO_2_ and CeO_2_ (>3.0 eV) do not, MnO_2_ has a lower band gap (ca. 2.16 eV), which could absorb light with λ > ca. 570 nm. To study this, we have measured film thickness while using profilometry, which shows that the TiO_2_ films are 0.40–0.50 μm, the CeO_2_ films are slightly thinner (0.15–0.30 μm), whilst the MnO_2_ films are the thinnest (0.10–0.15 μm), although they also contain occasional particles up to 0.30 μm, which may act as light scattering particles.

Further analysis of Figure 6 supports these assertions by considering the conduction and valence band energies of the various metal oxide scaffolds and the CH_3_NH_3_PbBr_3_ absorber [14,24,25,26]. Firstly, when comparing TiO_2_ and CeO_2_, the conduction band edges are in the same place (−4.0 eV). This is 0.62 eV more negative than the conduction band edge of CH_3_NH_3_PbBr_3_ and so effective electron injection would be expected into both TiO_2_ and CeO_2_. The open circuit voltage would also be expected to be similar while assuming similar Fermi levels for both metal oxides and this is what is measured in these devices (V_oc_ ≈ 0.9 V, Table 1). However, the J_sc_ for TiO_2_ is much higher than for CeO_2_, which suggests that either there is a poor CeO_2_-perovskite interface that limits injection or there are surface trap states on the CeO_2_ which recombine with the excited state electrons. Figure 6 also clearly shows the significant energy level mismatch (2.42 eV) between the conduction bands of MnO_2_ (−5.80 eV) and CH_3_NH_3_PbBr_3_ (−3.38 eV). Such is the offset, the valence band of CH_3_NH_3_PbBr_3_ is much closer to the conduction band of MnO_2._ As such, electron injection from the CH_3_NH_3_PbBr_3_ absorber into the MnO_2_ conduction band should result in a decrease of V_oc_ for these devices. However, as discussed previously, the increased V_oc_ that is observed suggests that the MnO_2_ is functioning as a scaffold rather than an electron transport layer, as was reported for alumina scaffolds in PSC devices [23].

These assertions are further backed up by the data for devices that were fabricated while using mesoporous scaffolds sintered at lower temperature (325 °C). None of the devices generated any photocurrent, which resulted in device efficiencies of ca. 0%. For CeO_2_, although the conduction band edge would be expected to be significantly shifted by a lower sintering temperature, the poor J_sc_ can be explained, because residual carbon would still be expected to be present within the mesoporous films as complete burnout would not be expected until ca. 375 °C (Figure 1). By comparison, for MnO_2_ complete carbon burnout would be complete by ca. 275 °C. However, a partial reduction on Mn^4+^ would create reduced manganese ions, which, in turn, would create new defect electronic states in the scaffold. Thus, instead of charge passing through the CH_3_NH_3_PbBr_3_, it is assumed that it recombines with these trap states, resulting in zero efficiency devices. 

UV-visible spectra have been measured to study the light harvesting of the perovskite-metal oxide films. As such, Figure S1 shows absorbance spectra for CH_3_NH_3_PbBr_3_ films that were deposited onto FTO-coated glass coated either with TiO_2_, CeO_2_ or MnO_2_ scaffolds. Each spectrum shows three key features; an absorption edge (I), a peak around 550 nm and a shoulder around 600 nm for the tribromide perovskite. For the TiO_2_ sample, the absorption edge is at 350 nm with peaks II and III at 544 nm (2.28 eV) and 601 nm (2.07 eV), respectively. For the CeO_2_ sample, these features shift to I = 335 nm, II = 549 nm (2.26 eV), and III = 613 nm (2.03 eV). For the MnO_2_ sample, the features are at I = 328 nm, II = 547 nm (2.27 eV), and III = 609 nm (2.04 eV). The data suggest that the MnO_2_ layer is sufficiently transparent that it does not limit light harvesting relative to the TiO_2_ or CeO_2_ scaffolds. We have also run ATR-infrared spectroscopy to confirm the absence of organic residues on the sintered metal oxide scaffolds (Figure S2).

**Figure 7:**
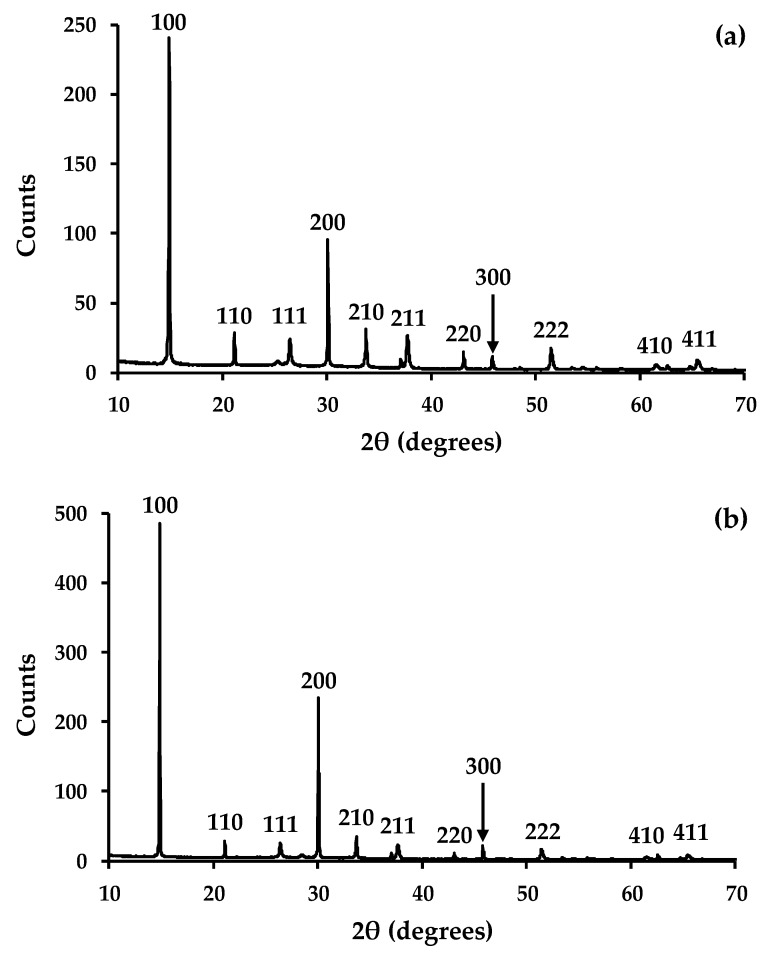
X-ray diffraction data of CH3NH3PbBr3 deposited on a TiO_2_ scaffold layer (**a**) as made and (**b**) after 24 h exposure to UV light and 70% relative humidity.

The potential influence of the metal oxide on perovskite crystallisation has also been studied using X-ray diffraction. The data (Figure 7a) show diffraction peaks for the 100, 110, 111, 200, 210, 211, 220, 300, 311, 222, 320, 321, 400, 410, and 411 planes of CH_3_NH_3_PbBr_3_ perovskite phase. In all of the samples, the preferred orientation is observed along the 100 and 200 planes. However, for the “as prepared” films, the intensities of the diffraction peaks that were observed for the 111, 210, 211, 220, 300, 311, 222, 320, 321, 400, 410, and 411 planes are higher relative to the 100 and 200 planes for CH_3_NH_3_PbBr_3_ material deposited on TiO_2_ when compared to the perovskite that was deposited on either CeO_2_ (Figure S3) or MnO_2_ (Figure S4). This suggests that the preferred orientation is less pronounced for the perovskite that was deposited on TiO_2_ when compared to CeO_2_ or MnO_2_.

The films were also exposed to a combination of UV light and relative humidity for 24 h to study perovskite stability on the different metal oxide scaffolds. Over this time period, the XRD data show for the CH_3_NH_3_PbBr_3_ films deposited on either CeO_2_ (Figure S3) or MnO_2_ (Figure S4) show that these materials do not change under these conditions. By comparison, the data for CH_3_NH_3_PbBr_3_ on TiO_2_ (Figure 7b) show a slight increase in the preferred orientation along the 100 and 200 planes, but no evidence for any degradation of the perovskite. We have further tested film stability by exposing all of the films to ambient conditions for a further week and the XRD data (Figure S5) also show no changes to the CH_3_NH_3_PbBr_3_ perovskite material. Whilst we have previously observed enhanced perovskite stability for organolead tri-iodide perovskites on alumina scaffolds [27], we ascribe this film stability to the inherently chemical stability of tribromide perovskites. However, these data do suggest that the different metal oxide scaffolds do not have a negative effect on film stability.

## 4. Conclusions

From a materials perspective, this paper shows that producing mesoporous metal oxide scaffolds while using lower sintering temperatures is possible using CeO_2_ or MnO_2_. However, residual carbon in CeO_2_ or new energy levels created from reduction of Mn^4+^ in MnO_2_ do limit solar cell efficiency. However, importantly, the data do suggest that, if energy level mismatches are sufficient, organolead perovskites can still work efficiently with low band gap metal oxides, such as MnO_2_, in a similar manner to the way they operate with large band gap insulators, such as Al_2_O_3_. Stability data suggest that different metal oxide scaffolds do not limit the inherent chemical stability of organolead tribromide perovskites. This opens up the possibility that it might be possible to use a much wider range of metal oxides as mesoporous scaffolds for perovskite solar cells than previously thought to be possible.

## Figures and Tables

**Table 1:** I-V data for CH_3_NH_3_PbBr_3_ PSC devices with mesoporous films sintered at 550 °C. The direction of voltage sweep is shown as oc-sc = open circuit to short circuit and vice versa.

Sample	PCE (%)	V_oc_ (V)	J_sc_ (mA cm^−2^)	FF
TiO_2_ oc-sc	3.8	0.95	8.91	0.45
TiO_2_ sc-oc	2.9	0.81	7.45	0.47
CeO_2_ oc-sc	0.9	0.91	2.55	0.37
CeO_2_ sc-oc	0.8	0.87	2.67	0.34
MnO_2_ oc-sc	3.9	1.34	7.50	0.38
MnO_2_ sc-oc	2.6	1.26	5.89	0.35

## Supplementary Materials

The following are available online at https://www.mdpi.com/1996-1944/13/4/949/s1, Figure S1: UV-visible spectra of CH_3_NH_3_PbBr_3_ perovskite films deposited onto (a) TiO_2_ scaffold, (b) CeO_2_ scaffold, and (c) MnO_2_ scaffold, Figure S2: ATR-infrared spectra of 500 °C sintered films of (a) TiO_2_ scaffold, (b) CeO_2_ scaffold, and (c) MnO_2_ scaffold, Figure S3: XRD data of CH_3_NH_3_PbBr_3_ films deposited on CeO_2_ scaffold; (top) as deposited and (bottom) after 24h exposed to UV and 70% relative humidity, Figure S4: XRD data of CH_3_NH_3_PbBr_3_ films deposited on MnO_2_ scaffold; (top) as deposited and (bottom) after 24h exposed to UV and 70% relative humidity, Figure S5: XRD data of CH_3_NH_3_PbBr_3_ films deposited on (top) TiO_2_ scaffold, (middle) CeO_2_ scaffold, and (bottom) MnO_2_ scaffold after exposure to ambient conditions for 1 week.
