# Supplementary material for: Metal Oxide Oxidation Catalysts as Scaffolds for Perovskite Solar Cells
Source: Materials (Basel). 2020 Feb 20;13(4):949. doi: 10.3390/ma13040949 (PMC7079644; doi:10.3390/ma13040949)
Supplement: Supplementary file 1 [file materials-13-00949-s001.pdf]

*materials*

SUPPLEMENTARY

# Metal Oxide Oxidation Catalysts as Scaffolds for Perovskite Solar Cells

**Peter J. Holliman *, Arthur Connell, Eurig W. Jones and Christopher P. Kershaw**

College of Engineering, Bay Campus, Swansea University, SA1 8EN Swansea, UK; arthur.connell@swansea.ac.uk (A.C.); eurig.w.jones@swansea.ac.uk (E.J.); c.p.kershaw@swansea.ac.uk (C.P.K.)

* Correspondence: p.j.holliman@swansea.ac.uk

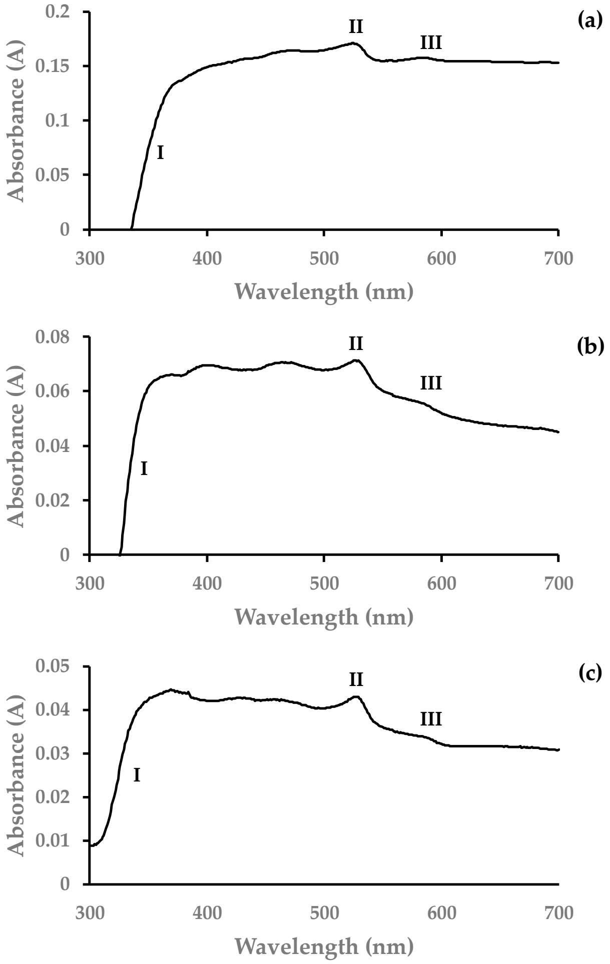


**Figure S1.** UV-visible spectra of $CH_3NH_3PbBr_3$ perovskite films deposited onto (**a**) $TiO_2$ scaffold, (**b**) $CeO_2$ scaffold, and (**c**) $MnO_2$ scaffold.

We have also run ATR to confirm the absence of organic residues on the sintered metal oxide scaffolds (please see Figure S2 below).

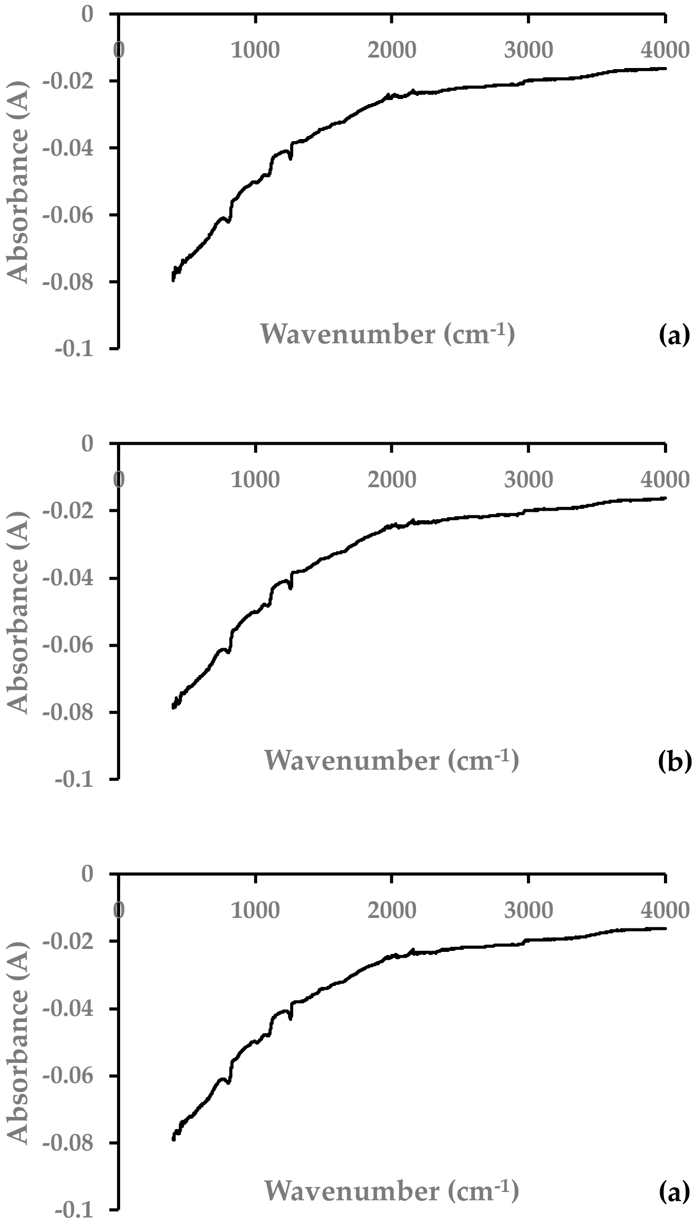


**Figure S2.** ATR-infrared spectra of 500 °C sintered films of (**a**) $TiO_2$ scaffold, (**b**) $CeO_2$ scaffold, and (**c**) $MnO_2$ scaffold.

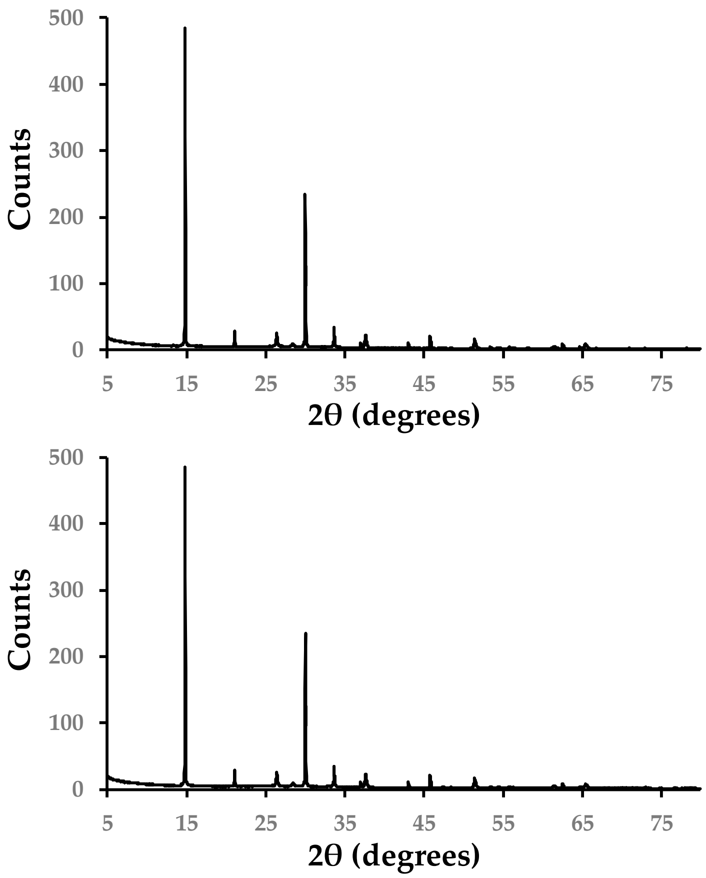


**Figure S3.** XRD data of $CH_3NH_3PbBr_3$ films deposited on $CeO_2$ scaffold; (top) as deposited and (bottom) after 24 h exposed to UV and 70% relative humidity.

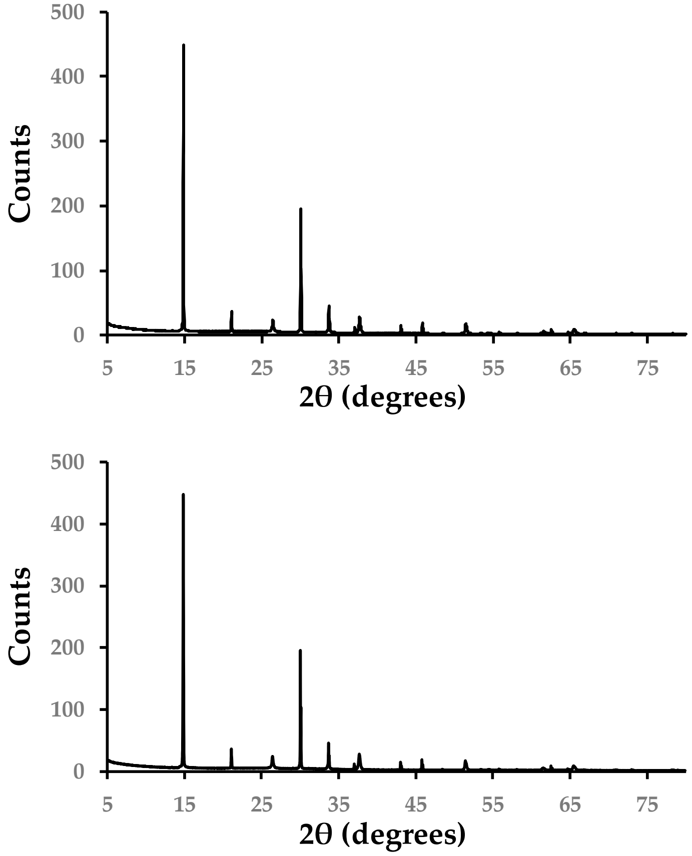


**Figure S4.** XRD data of $CH_3NH_3PbBr_3$ films deposited on $MnO_2$ scaffold; (top) as deposited and (bottom) after 24 h exposed to UV and 70% relative humidity.

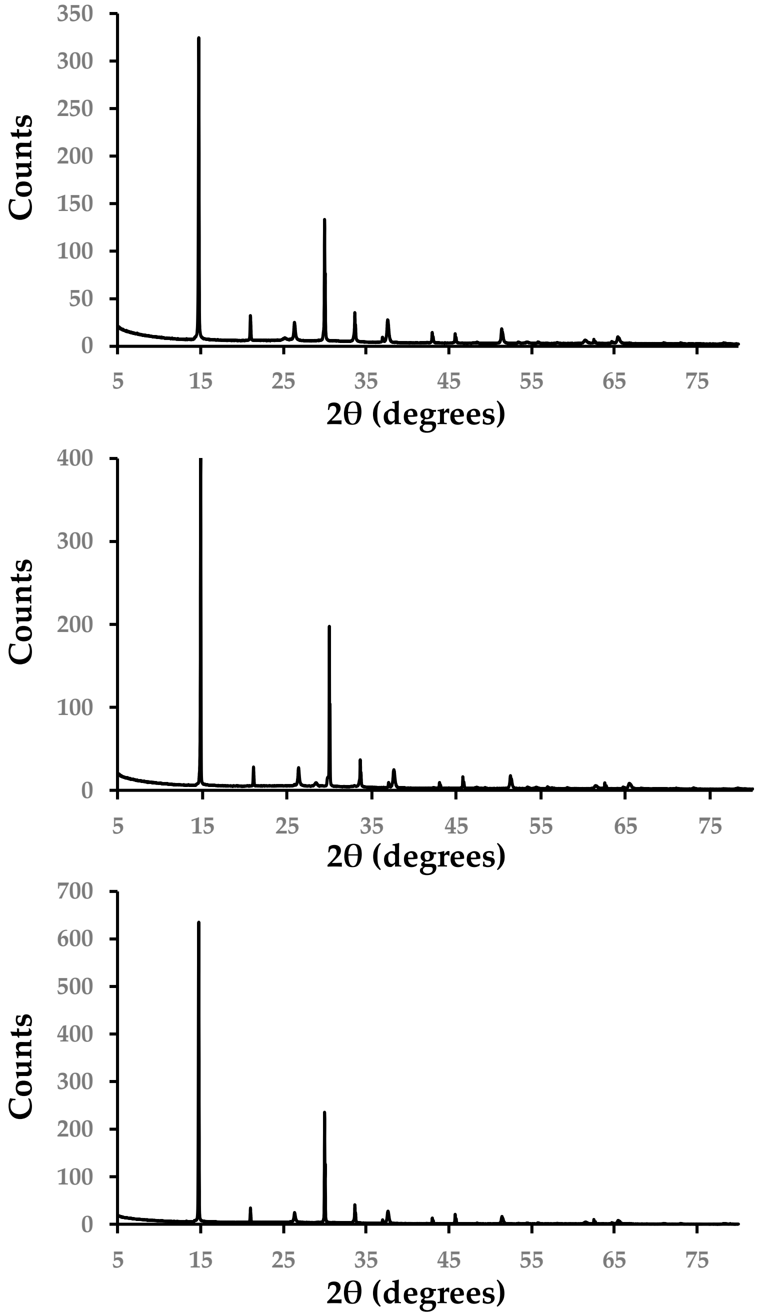


**Figure S5.** XRD data of $CH_3NH_3PbBr_3$ films deposited on (top) $TiO_2$ scaffold, (middle) $CeO_2$ scaffold, and (bottom) $MnO_2$ scaffold after exposure to ambient conditions for 1 week.
